# Patient Perspectives on a Patient‐Facing Tool for Lung Cancer Screening

**DOI:** 10.1111/hex.14143

**Published:** 2024-07-11

**Authors:** Victoria L. Tiase, Grace Richards, Teresa Taft, Leticia Stevens, Christian Balbin, Kimberly A. Kaphingst, Angela Fagerlin, Tanner Caverly, Polina Kukhareva, Michael Flynn, Jorie M. Butler, Kensaku Kawamoto

**Affiliations:** ^1^ Department of Biomedical Informatics University of Utah Salt Lake City Utah USA; ^2^ Department of Biomedical Engineering University of Utah Salt Lake City Utah USA; ^3^ Department of Communication and Huntsman Cancer Institute University of Utah Salt Lake City Utah USA; ^4^ Department of Population Health Sciences University of Utah Salt Lake City Utah USA; ^5^ Department of Internal Medicine University of Michigan Ann Arbor Michigan USA; ^6^ Departments of Internal Medicine and Pediatrics, Community Physicians Group University of Utah Health Salt Lake City Utah USA

**Keywords:** cancer screening tests, decision aid, informatics, lung cancer, patient preferences, shared decision‐making

## Abstract

**Background:**

Individuals with high risk for lung cancer may benefit from lung cancer screening, but there are associated risks as well as benefits. Shared decision‐making (SDM) tools with personalized information may provide key support for patients. Understanding patient perspectives on educational tools to facilitate SDM for lung cancer screening may support tool development.

**Aim:**

This study aimed to explore patient perspectives related to a SDM tool for lung cancer screening using a qualitative approach.

**Methods:**

We elicited patient perspectives by showing a provider‐facing SDM tool. Focus group interviews that ranged in duration from 1.5 to 2 h were conducted with 23 individuals with high risk for lung cancer. Data were interpreted inductively using thematic analysis to identify patients' thoughts on and desires for a patient‐facing SDM tool.

**Results:**

The findings highlight that patients would like to have educational information related to lung cancer screening. We identified several key themes to be considered in the future development of patient‐facing tools: *barriers to acceptance, preference against screening* and *seeking empowerment*. One further theme illustrated *effects of patient–provider relationship* as a limitation to meeting lung cancer screening information needs. Participants also noted several suggestions for the design of technology decision aids.

**Conclusion:**

These findings suggest that patients desire additional information on lung cancer screening in advance of clinical visits. However, there are several issues that must be considered in the design and development of technology to meet the information needs of patients for lung cancer screening decisions.

**Patient or Public Contribution:**

Patients, service users, caregivers or members of the public were not involved in the study design, conduct, analysis or interpretation of the data. However, clinical experts in health communication provided detailed feedback on the study protocol, including the focus group approach. The study findings contribute to a better understanding of patient expectations for lung cancer screening decisions and may inform future development of tools for SDM.

## 1 Introduction

Lung cancer is the leading cause of cancer‐related deaths among people worldwide [1]. Lung cancer screening can play a vital role in decreasing lung cancer deaths by identifying the disease at an earlier, more treatable stage [2]. By catching lung cancer before it has a chance to advance, screening offers a critical opportunity to intervene when it is most manageable [3]. Using low‐dose computed tomography (LDCT) scans, high‐risk individuals, particularly individuals who smoke(d) heavily, can undergo regular screening [4]. When LDCT screening detects lung cancer early, it often allows for timely treatment, such as surgery or radiation therapy, which can lead to reduced lung cancer mortality [5, 6]. Moreover, as treatment options continue to evolve, early detection through screening becomes even more crucial in the fight against lung cancer, ultimately reducing the number of lives lost [4]. Despite its potential to detect lung cancer in high‐risk populations, lung cancer screening has been historically underutilized [7, 8].

Shared decision‐making (SDM) is instrumental because it empowers individuals to make informed choices about their healthcare in collaboration with their clinicians [9]. By involving patients in the decision‐making process, healthcare providers can ensure that decisions align with a patient's values, preferences and individual risk factors [10]. This collaborative approach fosters trust between patients and healthcare professionals, enhances patient satisfaction and ultimately leads to more effective and patient‐centred care [11]. Furthermore, SDM encourages patients to take an active role in managing their health, leading to better adherence to recommended screening protocols [11].

Many national and international organizations consider SDM to be standard when it comes to clinical practice regarding decision‐making about lung cancer screening. The US Preventive Services Task Force (USPSTF) recommends that lung cancer screening should include SDM [3]. The Centers for Medicare and Medicaid Services (CMS) provide coverage for eligible patient visits only when SDM patient decision aids are used [12]. An expert panel from the American College of Chest Physicians (CHEST) found that LDCT screening for lung cancer is a beneficial procedure but the trade‐offs between benefit and harm are patient‐sensitive [13, 14]. To meet standards and increase the use of SDM, electronic decision aids that incorporate risk prediction calculators are becoming increasingly preferred in lung cancer screening SDM procedures [15].

In the context of lung cancer screening, the balance between benefits and risks can be challenging [4]. Lung cancer screening carries the risk of false‐positive results and the associated stress of further testing or invasive follow‐up procedures [4, 16]. Moreover, the screening benefit varies greatly across patients, due in part to lung cancer risk and life expectancy, making it challenging to identify candidates for screening [17, 18]. Decision aids, in the form of paper handouts, videos and/or electronic‐based tools (e.g., websites, apps), can provide additional information about the decision to be made along with the options and how they may relate to patient preferences [19]. The use of decision aids in the lung cancer screening process can enable an informed conversation between patient and provider to determine whether screening is appropriate for a particular individual, ensuring that the benefits outweigh the potential harms [20].

**Figure 1:**
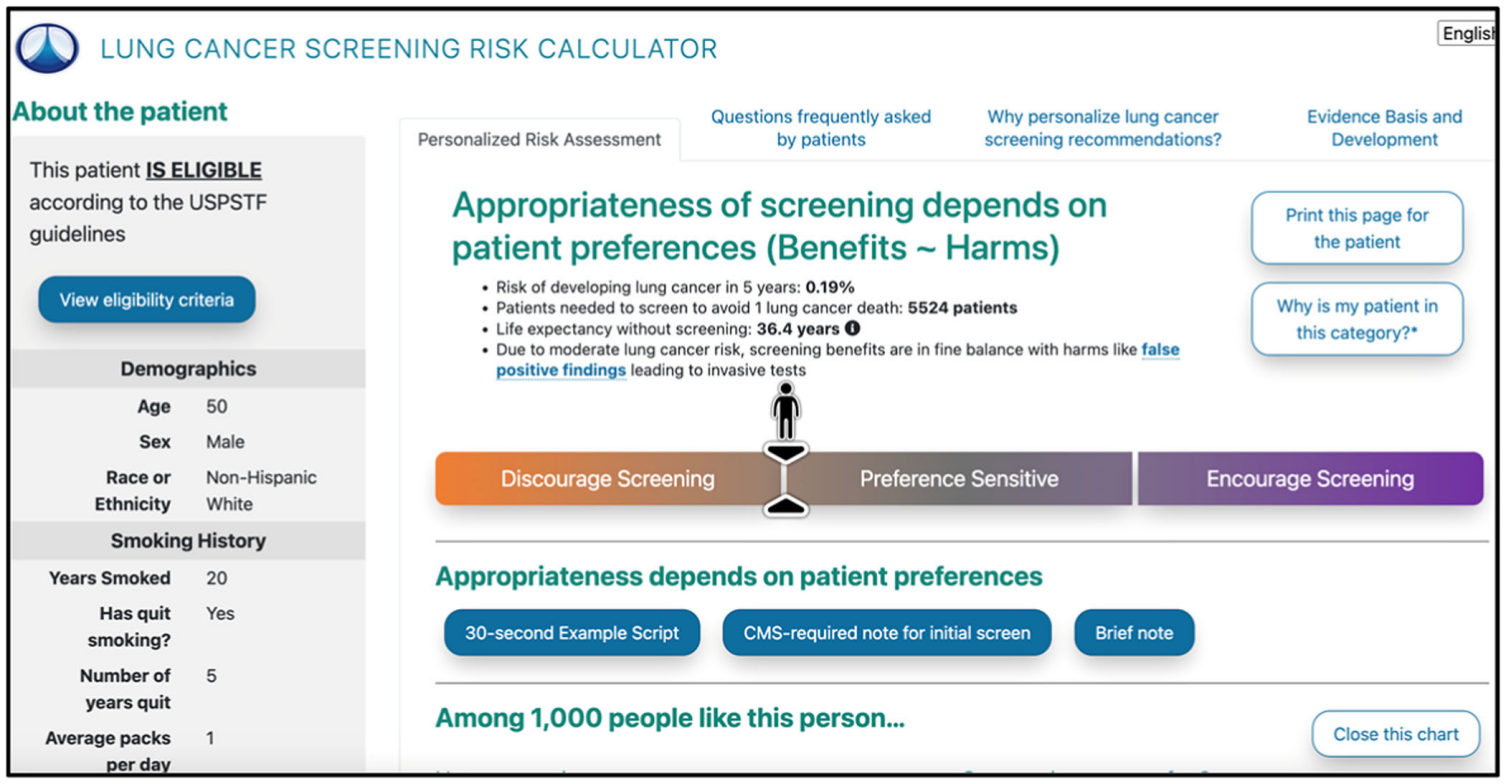
Decision precision lung cancer screening risk calculator (screenlc.com).

Previous work has demonstrated that web‐based decision aids for lung cancer screening are likely useful to patients and support the development of technology for use in the clinical setting [21, 22, 23]. One example of a tool designed for lung cancer screening SDM is Decision Precision, also known as ScreenLC (due to its availability at screenlc.com), an electronic web‐based decision aid utilized by providers across the nation, including at University of Utah Health [24]. This application allows the provider to view a patient's lung cancer risk profile and includes a patient‐specific risk calculator (Figure 1). Decision Precision is reviewed by the provider and patient during an in‐person visit. A key challenge with the approach is the time that it takes to use in busy clinical settings. Even when integrated with electronic medical records, providers often do not have the time to conduct SDM [25, 26]. Patient‐facing educational tools could therefore potentially facilitate SDM and lung cancer screening by empowering patients with this information outside of busy clinical visit settings. However, in the Decision Precision process, patients are not provided a patient‐facing decision aid before the visit, leaving the patient without advance access to information about their risks and benefits.

In this study, we sought to assess patient interests and thoughts on having access to a web‐based decision aid for lung cancer screening before an appointment. We leveraged the existing Decision Precision tool to inform the design and development of future decision aids that eligible patients would have the option to access before and after provider visits. Our vision was that the tool would have the ability to provide effective and succinct personalized information, encourage questions during the SDM process between the patient and a provider and be appropriate for patients with low levels of health literacy [27, 28]. The purpose of this study was to explore patient perspectives related to the usefulness of the current provider‐focused tool and understand the information needs required for a patient‐facing version to facilitate lung cancer screening SDM.

## 2 Methods

We used a qualitative descriptive approach based on semi‐structured focus groups with participants identified as eligible for screening [29]. Qualitative description suited the purpose of the study, addressing limitations in the description and conceptualization of decision aids used by individuals for lung cancer screening SDM, and capturing the who, what and where of experiences to gain insights from informants [30]. This approach allowed the research team to (1) gain intimate knowledge of patients' interest in lung cancer screening education to facilitate SDM and (2) their perspectives on using a patient‐facing tool.

### 2.1 Recruitment

Focus group recruitment included direct outreach through collaboration with the University of Utah Huntsman Cancer Institute's Health Outcomes and Population Equity (HOPE) programme for tobacco cessation and the University of Utah Community Collaboration & Engagement Team (CCET). Individuals in the HOPE programme database who met inclusion criteria were contacted by email. We also distributed flyers via online platforms, in person at University of Utah Health clinics and through ResearchMatch (Vanderbilt University, Nashville, TN). Interested individuals were invited to fill out a screening survey to assess eligibility.

The inclusion criteria were developed in accordance with 2021 screening guidelines recommended by the United States Preventive Services Task Force (USPSTF) [3]. The inclusion criteria for this study included age range of 50–80 years, smoking history of 20 pack years or more and an individual who smokes or had quit within the past 15 years. Individuals with a past or current lung cancer diagnosis were excluded. Due to constraints of the focus group methods, potential participants were excluded if they did not self‐identify as ‘very comfortable’ or ‘somewhat comfortable’ with video conferencing. Individuals were also excluded if they did not complete the screening survey in its entirety.

### 2.2 Data Collection

This study was approved by the University of Utah Institutional Review Board. Data were collected in two stages. First, interested individuals who met study criteria were referred for initial consent and screening through the study website and by email. Once eligibility was confirmed and consent was completed, individuals were asked to complete a demographic survey hosted by REDCap [31, 32]. Those who agreed to be part of focus group interviews were contacted for a recorded video conference.

### 2.3 Focus Group Procedures

The focus groups took place in the second half of 2022 and early 2023 using the Zoom video conferencing tool (Zoom Video Communications Inc) and ranged in duration from 1.5 to 2 h. After introductory remarks and consent review, participants were given a short presentation about the Decision Precision and provided some background information about lung cancer and low‐dose CT screening. Focus group participants were shown the Decision Precision, with a demonstration of the personalized risk calculator. The potential for adaptation to a patient‐facing tool was described. Focus group interviews included questions such as the following: (1) What are your thoughts about talking with your doctor about lung cancer screening? (2) What information in the application would you want to see before visit with your provider? (3) How would this tool be useful to you as a patient (Supporting Information)? We also asked participants for suggestions to make the application easier to use. The focus group facilitator script was developed using the framework found in the ‘Sample Focus Group Moderator's Guide’ in Making Health Communication Programs Work along with health communication experts at the University of Utah [33]. Follow‐up questions were designed to elicit details about what information was needed to make a screening decision and their expectations regarding SDM. We conducted focus groups until we reached saturation, and no new ideas were presented [34]. Upon completion of the focus group interviews, participants were emailed a $50 gift certificate. Zoom audio recordings were downloaded and securely transmitted to a transcription service. Transcripts were anonymized and placed in secure storage, following best practices for qualitative research reproducibility.

### 2.4 Data Analysis

We used Braun and Clarke's inductive thematic analysis to analyse the data, given its suitability as an approach to elicit views, opinions, experiences or values [35]. Using Dedoose Version 9.0.17, a qualitative data analysis web application, transcripts were uploaded and text segments were jointly coded by four members of the research team [36]. Deductively generated codes congruent with the lung cancer screening SDM process were applied for initial coding of the first focus group. Subsequent codes were inductively generated, using open codes developed through group consensus coding as more data were analysed. Both inductive and deductive codes were defined and elaborated throughout the analysis process. Definitions facilitated consistent coding across the team. Regular coder‐team meetings focused on continued refinement of the codebook and the exclusion of nonfunctional codes until all focus groups were analysed.

We generated statistics for the participant sociodemographic factors to quantitatively describe the sample. At the conclusion of coding, we assembled categories of lung cancer screening information needs. These categories were initially developed by clustering similar codes together. We then worked abductively between the data and the literature to achieve the best fit of the data into categories. Using this technique allowed us to progress from coded lists based on the transcripts, then to categories based on those codes and finally to an organized set of themes that were situated relative to lung cancer screening and SDM.

We addressed rigour in the research process through systematic methods of code development and application, team‐based analysis, group consensus and reflexivity. Weekly consensus meetings with the research team included extensive discussion to address discrepancies and consistencies in coding and the representation of conceptual results.

Members of the research team were trained in health informatics or were healthcare professionals familiar with information needs related to decision aids. These experiential assets added to our ability to reflexively interpret the data, valuing both our meaningful insights and acknowledging our biases through team‐based discussion [37].

## 3 Results

Twenty‐three individuals met inclusion criteria through the screening survey and were interviewed over four focus groups, with five to six individuals per focus group. The mean age of the participants was 58 years and over 61% were female. A summary of participant characteristics is provided in Table 1.

**Table 1:** Focus group participant demographics.

Characteristic	% (*N*)
Age 50–59	61 (14)
Age 60–69	35 (8)
Age 70 +	4 (1)
Female	61 (14)
Male	35 (8)
Nonbinary	4 (1)
White	74 (17)
Hispanic	13 (3)
Non‐Hispanic Black	13 (3)
Annual household income ≥ $50,000	61 (14)
Annual household income < $50,000	39 (9)

Overall, we found that many participants were interested in learning more about lung cancer screening. They expressed a desire to use a lung cancer screening educational tool before a visit and during a visit in consultation with a provider. Participants also provided perspectives on how they would like to receive and interact with the information.

Four key themes were identified as comprising the shared perspectives of participating individuals at risk for lung cancer in relation to lung cancer screening: *barriers to acceptance*, *preference against screening, seeking empowerment* and *effects of patient–provider relationship*. In addition to the overarching themes, we generated a list of functionality requests and user interface design suggestions. The list of requests and suggestions along with themes and subthemes are presented below.

### 3.1 THEME 1: Barriers to Acceptance

Although the study intended to focus exclusively on patient interests related to the use of a web‐based decision aid for lung cancer screening, a repeated phenomenon noted by researchers during interviews was that participants consistently refocused the conversation back to concerns that may hinder acceptance of lung cancer screening such as their intentions regarding smoking cessation and knowing whether one has lung cancer. This points to the complex relationship that society has with technology and health information, ranging from exclusion and challenges to varying levels of acceptance and rejection. The subthemes included strong emotions when discussing lung cancer risk centring around fear, hopelessness and determination to preserve one's autonomy regarding the choice to smoke.

Some participants expressed that they did not want to know if they had lung cancer because they were not planning to change their behaviours regardless of the result. Participants also expressed a fear of positive screening results and spent time deliberating whether they would want to know or not. One participant shared:

I mean, it's a scary thought to have to do that because I think, as a smoker, you're always afraid that you're going to get lung cancer. And sometimes the decision is, ‘Do I want to know, or do I not want to know?’ which I think always plays in the back of your head. But I think, for me, I'd rather know early so that I can have it taken care of, hopefully, and not have to get it at the end when nothing can be done.

Another participant explained that they were not interested in knowing about lung cancer:

When I was smoking, I would have just said, ‘I don't care. This is a passive form of suicide. Go away. Leave me alone.’ But I haven't smoked for 12 years. And there was a period where I didn't have any health insurance. And then again, if there's something wrong with me, I don't want to know, because I can't do anything about that.

Looking at their risk for cancer evoked emotions of depression and anxiety in some participants, which in turn led to a desire for smoking. Participants provided comments such as:

It was depressing…yeah, I read it…it was, yeah…depressing.

And another expressed:

You'll smoke more cigarettes waiting on the results…once you find out, you'll be smoking three cartons a day.

### 3.2 THEME 2: Preference Against Screening

*Preference against screening* represents how participants described preconceived notions as to why they do not need to get screened despite viewing their level of risk. Participants with high risk of lung cancer described multiple approaches to rationalize their thoughts ranging from (1) going by feel, to (2) lack of family history and (3) ‘worth it’ beliefs. All approaches balance beliefs and experiences, resulting in strong screening preferences. The following quotes illustrate participant beliefs that they would sense in their body if something was wrong as well as their greater trust in those inner feelings over medical test results. An example of this is as follows:

I'm hesitant only because I've had screenings in other areas where I've gotten false positives. So for me, relying on how I feel, how I breathe, if it affects me is more important.

Another participant expressed:

I'd rather just kind of rely on how my body feels and what my physician says, and that's what I do.

And another participant explained:

What I saw is that there's a slim chance of being cured, so I wouldn't take the test, unless my own physical self told me that there was something terribly wrong because you can feel it.

A number of participants described basing their decision on the cancer status of family members who smoked cigarettes.

As far as having a cancer running through my family, it's just not there. What are the odds that I'm 45 years in smoking—I mean, I've been smoking some good cigarettes 45 years. And I'm okay.

Even my grandfather who was a physician smoked, right, because back in the turn of the last century, definitely nobody knew it was bad. And still nothing on lung cancer within the family.

Others indicated ‘worth it’ beliefs, suggesting that the benefits of smoking outweigh the risks.

At the end of the day, here's my take on it. We're all going to die from something.

So I just decided today that I only live once; I'm gonna eat what I want to, and if I go into diabetes, so be it. And that's the way I feel about smoking. I know what the risks are. So if I develop lung cancer, so be it. I'm 62, and I'm gonna live my life. If I want to smoke, I'm gonna smoke.

### 3.3 THEME 3: Seeking Empowerment

Empowerment was perceived as a facilitator of tool use and, overall, many participants stated that they would like to use a tool like Decision Precision because the information would empower them in making decisions about their healthcare. Aspects of empowerment expressed included access to information, the ability to say no after receiving information and having control of their health data.

Participants described having access to data and information as important to their decision‐making, with some pointing out that simply having the information did not necessarily mean that they would choose screening. Others desired a ‘full’ picture of their health instead of a disease‐by‐disease view so that all screenings could be addressed together. One participant explained:

… they didn't have the knowledge that they have today. So for me, the knowledge is key, and if I have the knowledge that I do have something, then I can visit the appropriate medical professionals and do whatever treatment I have to do to survive.

Another participant noted:

… it's a good way, a good tool to have to engage in your care, learn about your care, and what's going on and get a better possibly understanding. And even if, let's say, I ran through it and I didn't understand some things, I can say to my provider, ‘Hey, what does this all mean? What does this mean for me?’ So it's a great way to engage with your own care.

And another participant wanted as much information as possible:

I'm an information addict anyway, so I would like to look at that before making my decision, should I get it done or not. I would find it valuable. I don't know that I would base my decision to get the screening done off of that, but the more information, the way I see it, is—knowledge is power. I just like to have all the information I can get on anything.

Technology was seen as a positive adjunct by some participants, who indicated that they would use it if it was available. Participants did not express any concerns with the tool replacing face‐to‐face care.

I would want to interact with it. I think I'm pretty good technologically, so if I saw something there on my portal, I would definitely go into it to check it out, just because I'm always all up in the portal, in MyChart, in my messages, in my medications and stuff like that. I'm just proactive in that way. So if I saw a tool like that, I would definitely go in to see what it was all about and to see what conclusions I might get from it that can help me with my next doctor's visit or just a phone call with my doctor.

Participants expressed concerns regarding the accessibility of on‐line patient‐facing educational tools like Decision Precision for various reasons including age, disability or limited infrastructure availability (e.g., limited internet access in rural or underserved areas). One participant explained:

I think it just depends on how comfortable people are with the internet and those type of tools … but for some other people in a certain age group who are not used to … don't have a smartphone or don't have a laptop or a computer, for those people, it would be difficult.

Participants suggested modifications to support tool access by those with visual and hearing impairments or others with limited English proficiency since the Screen LC tool was only available in English at the time that focus groups were conducted.

‘Usually, they have like a both in Spanish and English and people can choose either one in Spanish or English…It's very useful …’. However, others were concerned with having control over their health data and the ability to make changes and updates to their data. The prospect of providers adding and editing data without their input was not acceptable.

But I also think you should be able to … update it and it stays updated, not like the physician doesn't go and update it without your permission. There has to be somebody who has the actual control over that data and whether they change it and you say, ‘No. I don't want you to change it to that. I have my own rights to what is in that data.’ There's got to be some kind of ownership of the data, and I think it's the patient who should have the control.

Another participant expressed frustration with data ownership and the ability to contribute to what is in the medical record:

Who has the control of that data? I think the patient needs to have more input.

### 3.4 THEME 4: Effects of Patient–Provider Relationship

Participants identified that in some situations, their relationship with their primary care provider was a barrier to getting screened. Some reasons provided were that their provider was not proactive about screening, they did not feel comfortable bringing it up with their provider or they were not honest about their smoking history with their provider. Participants expressed shame as a reason why they or others like them would not want to discuss lung cancer screening with their provider. Stigma around smoking was a common topic and some participants even described feeling like ‘second‐class citizens’ because they smoke and that ‘smoking has become hush‐hush. You don't tell people that you're a smoker’. One participant explained:

I'm not honest with my doctors about how much I smoke and the frequency of my smoking. And basically that's because of paper trails and health insurance and different factors that play into who reads your medical records. But they do know I smoke. I do lie about how much I smoke.

In contrast, participants indicated that having regular contact with a primary care provider would be a facilitator to tool use. The importance of having a good relationship with a trusted provider that they have known for a long time was stressed. An example from one participant is:

I feel it's been very important for me to find the right provider that I can connect with and talk to because of my … background. But the provider that I have now is someone that I can really talk to and open up and talk to about whatever … it took me a very long time to find that person that I can talk to about whatever.

### 3.5 Functionality Requests and User Interface Design Suggestions

Participants provided suggestions for desired features and functionality in a patient‐facing lung cancer screening decision tool (Table 2). After a guided review of the provider‐facing tool, participants provided suggestions for the user interface that would make the tool ‘easy to use’. Most of the participants requested the ability to select their own viewing preference (numbers vs. dots in an icon array) and preferred having wording alongside the images. Other minor suggestions included the use of a pie chart format for the data presentation and better cues for clickable text links.

**Table 2:** Participant suggestions for tool features.

Feature	*N* (%)
Ability to update smoking history for accuracy	9 (39)
Overall health summary to view all health recommendations at one time	7 (30)
Amount of insurance coverage for screening	6 (26)
Place to add notes and concerns	3 (13)
Glossary of terms related to screening (e.g., pack‐years, screening, eligibility)	2 (9)
Alert for the annual screening	2 (9)
Patient testimonials	1 (4)

## 4 Discussion

This qualitative study aimed to understand factors related to perceptions of the use of a tool to support SDM for lung cancer screening. We found that many participants were interested in having information about lung cancer screening and that the use of technology to receive the information was acceptable, while others preferred not to think about their risk of lung cancer. In exploring the acceptability and value of a patient‐facing decision aid, four key themes were identified. The themes point to a deeper examination of the verbal and written strategies required to influence and empower individuals to make informed choices for screening.

Two of the themes, *barriers to acceptance* and *preference against screening*, can be considered together. In studies of risk perceptions of individuals who smoke(d), risk‐minimizing and self‐exempting beliefs are prevalent [38, 39]. Individuals seek congruence among their beliefs, attitudes and behaviours. According to cognitive dissonance theory, when there is inconsistency (or dissonance), people will attempt to resolve it through a rationalization of their thoughts and/or actions [40]. In the case of individuals who smoke, they widely accept that smoking is bad for them, and yet continue to do it [41]. They also hold various beliefs to minimize the reality of the harms caused by smoking to allow them to avoid engaging in the task of quitting [42]. These concepts also translated to lung cancer screening beliefs from our findings. Participant beliefs suggest that the benefits of smoking outweigh the risks and some would rather not know about having lung cancer. Participants were also unrealistically optimistic about their low risk for lung cancer and considered other risks of dying to be far greater. Our findings suggest that helping individuals with high risk for lung cancer may require strategies such as those used for smoking cessation that emphasize the benefits of screening as well as the reduced quality of life associated with a lung cancer diagnosis [43]. Due to the potential denial of the real risks of smoking among individuals who smoke, future work to explore differences in screening barriers between individuals who smoke and those who smoked in the past would also be worthwhile [44, 45].

As demonstrated in the responses related to the third theme, *seeking empowerment*, providers can support patient goals by providing personalized data and information and offering practical suggestions to help them engage in best practices for lung cancer screening through the use of decision tools. Similar to findings obtained by Carter‐Harris et al., tailored and personalized guidance was seen as a favourable aspect of the decision aid and has the potential to increase knowledge [46]. Patient portals have the potential to provide patients with information as well as the ability to update or correct information, but best practices for portal outreach on lung cancer screening are unknown [47]. A deeper exploration of two‐way portal communication for screening efforts may increase patient empowerment and support SDM processes. Incorporating smoking cessation with screening discussions may further support decision‐making [48, 49].

Patient beliefs and experiences with healthcare providers revealed a fourth theme of *effects of patient–provider relationship*. Distrust in providers appears to be a major barrier to using an electronic decision aid in SDM for lung cancer screening. A strong bond between healthcare providers and patients enhances the effectiveness of smoking cessation interventions, as patients are more likely to follow advice and engage in programmes that they trust [50]. Similarly, our findings indicate some anxiety around sharing smoking information and knowing the screening results. However, Shapira et al. found no difference in anxiety or worry with a lung cancer screening SDM decision aid but an increase in knowledge [51]. This suggests that motivation and monitoring by providers may help maintain the patient's commitment to lung cancer screening. Strategies for SDM that include emotional support from providers may prove helpful, as talking about lung cancer and smoking cessation are often emotionally challenging. Given these findings, a dyadic approach to tool development may reveal opportunities to overcome barriers [52]. It would also be worthwhile to investigate the privacy aspects of patient‐facing tools, e.g., those that are not integrated into the EHR, and their impact on trust [53]. Provider education on interpreting and communicating personalized patient risks for lung cancer screening should be explored.

Specific suggested tool features shared in this study can inform the design of SDM interventions that can be disseminated through patient education materials, patient portals and other cancer prevention resources. Given our focus group findings, we suggest the following considerations in the development of SDM decision aids for lung cancer screening:

1. Make the decision aid accessible outside of visit, for example, connecting to resources through the patient portal.
2. Provide personalized information on the benefits of screening, especially for those at highest risk.
3. Identify and proactively engage with those at highest risk, for example, use prompting on lung cancer screening eligibility through their preferred communication channel.

Future work that leverages participatory design in the development of patient‐facing shared decision aids can help in making healthcare decisions that are more aligned with the patients' values, preferences and lifestyles. This may lead to choices that patients are more comfortable with and committed to, improving adherence to treatment plans. Ultimately, involving patients in crafting SDM interventions can contribute to a greater sense of health, well‐being and support.

### 4.1 Strengths and Limitations

With focus groups, we were able to obtain rich, qualitative data by allowing participants to discuss lung cancer screening perspectives in depth, revealing detailed insights that might not emerge in structured surveys. We believe that the methodology is a strength of this work, particularly in an exploratory research stage. The major limitation of this work was the small sample size. However, the focus groups represented a diversity of perspectives, and we reached saturation in our focus group responses. Additionally, perspectives were only gathered from human subjects who could be contacted through accessible networks, which may have excluded target users who do not have access to technology or regular care. However, given that this work explored the development of a patient‐facing technology, a certain level of technological comfort was warranted. Lastly, only English‐speaking participants were included in the focus groups. Now that the tool has been translated beyond English, it would be helpful to repeat this study with individuals with limited English proficiency.

## 5 Conclusion

In summary, this study's findings support the case for development of educational tools to assist patients in making potentially life‐saving LDCT lung cancer screening decisions. We found that participants at risk for lung cancer would find an educational tool for patients to facilitate SDM acceptable. However, there are several issues that must be considered. The intention is that through the development of web‐based, mobile‐accessible SDM tools, we should be able to increase the utilization of lung cancer screening by eligible patients and ultimately decrease lung cancer mortality.

## Author Contributions

**Victoria L. Tiase:** formal analysis, data curation, writing–original draft, writing–review and editing, investigation. **Grace Richards:** data curation, formal analysis, writing–original draft, conceptualization, methodology, investigation, writing–review and editing. **Teresa Taft:** data curation, conceptualization, methodology, investigation, writing–review and editing. **Leticia Stevens:** data curation, formal analysis, investigation, writing–review and editing. **Christian Balbin:** data curation, formal analysis, investigation, writing–review and editing. **Kimberly A. Kaphingst:** methodology, conceptualization, writing–review and editing. **Angela Fagerlin:** writing–review and editing. **Tanner Caverly:** writing–review and editing. **Polina Kukhareva:** writing–review and editing. **Michael Flynn:** writing–review and editing. **Jorie M. Butler:** data curation, investigation, formal analysis, writing–review and editing. **Kensaku Kawamoto:** conceptualization, methodology, supervision, project administration, funding acquisition, writing–review and editing.

## Ethics Statement

Ethical approval was obtained from the University of Utah Institutional Review Board #00142266. Approval period: 16 July 2021 to 31 December 2024.

## Conflicts of Interest

The authors declare no conflicts of interest.

## Supporting information

Supporting information.

## Data Availability

The data that support the findings of this study are available on request from the corresponding author. The data are not publicly available due to privacy or ethical restrictions.
