# Supplementary material for: Patient Perspectives on a Patient‐Facing Tool for Lung Cancer Screening
Source: Health Expect. 2024 Jul 11;27(4):e14143. doi: 10.1111/hex.14143 (PMC11239535; doi:10.1111/hex.14143)
Supplement: Supplementary file 1 — Supporting information. [file HEX-27-e14143-s001.docx]

**Supplementary Material**

Focus Group Questions

1. What comes to mind when you think about lung cancer and lung cancer screening?
2. What are your thoughts about talking with your doctor about lung cancer screening?
3. What information in the application would you want to see before visit with your provider? How about after a visit with your provider?
4. How would this tool be useful to you as a patient?
5. What might be some barriers for you or individuals like yourself to using this tool?
6. What suggestions do you have to make the application easier to use/understand?
   1. What should be added for patients?
   2. What should be removed for patients?
7. What do you think of the risk calculator?
